# Investigation of the maxillary lateral incisor agenesis and associated dental anomalies in an orthodontic patient population

**DOI:** 10.4317/medoral.17767

**Published:** 2012-05-01

**Authors:** Mevlut Celikoglu, Hasan Kamak, Hanifi Yildirim, Ismail Ceylan

**Affiliations:** 1 Assistant Professor, DDS, PhD Department of Orthodontics, Faculty of Dentistry, Karadeniz Technical University, Trabzon, Turkey; 2 Assistant Professor, DDS, PhD Department of Orthodontics, Faculty of Dentistry, Kirikkale University, Kirikkale, Turkey; 3 Research Assistant, DDS, Department of Orthodontics, Faculty of Dentistry, Ataturk University, Erzurum, Turkey; 4 Professor, DDS, PhD, Department of Orthodontics, Faculty of Dentistry, Ataturk University, Erzurum, Turkey

## Abstract

Objectives: The aim of this study was to investigate the prevalence of maxillary lateral incisor (MLI) agenesis and associated dental anomalies as well as skeletal patterns in an orthodontic population, and then to compare it with the prevalence of these anomalies in the general population. Study Design: The material of the present study included the records of the 3872 orthodontic patients. The followings were recorded for each subject with the agenesis of MLI: Age, sex, unilateral or bilateral absence, anterior-posterior skeletal relationship of the maxilla and mandible, and presence of associated dental anomalies. The occurrence of these anomalies was compared with data previously reported for the general populations. Results: Of the 3872 patients examined, 94 were found to have agenesis of the MLI, representing a prevalence of 2.4 per cent, with females being more frequently observed. The most commonly found associated anomalies were ectopic eruption of maxillary canines and reduced or peg- shaped contralateral incisor with the frequencies of 21.3 per cent and 20.2 per cent respectively. Conclusions: Patients with agenesis of MLI showed a significantly higher prevalence of skeletal Class III malocclusion compared with the general population. The prevalence of ectopic eruption, transposition, and transmigration of the maxillary canine and reduced or peg- shaped MLIs were significantly increased.

** Key words:**Hypodontia, missing laterals, associated dental anomalies.

## Introduction

Tooth agenesis, the congenital absence of one or more primary or permanent teeth, is one of the most frequently observed dental anomalies in children (1). The data for tooth agenesis, excluding the third molars, in both genders varies between 0.3 per cent and 11.3 per cent (2-4). Higher frequencies of tooth agenesis have been reported in females than in males (1,5-7). Although local, systemic and genetic factors have been implicated in the aetiology of this anomaly, the extent to which genetic and/or environmental factors are involved remains unknown (8,9). Dental anomalies such as peg-shaped incisors, taurodontism, transposed teeth, supernumerary teeth, and ectopic eruption may occur in subjects with tooth agenesis (10-14).

Maxillary lateral incisor (MLI) is one of the more frequently missing teeth after the third molars (1,4,6,7,15,16). Previous studies have shown that this tooth is the second most frequently missing tooth after the third molars. Early recognition of a tooth agenesis is helpful in order to provide adequate treatment and prevent a developing malocclusion (9). Orthodontic treatment may involve closure of excess space or opening a space in the arch for a prosthetic replacement or implant (4). There is remarkably little information in the literature on the prevalence of other dental anomalies and the skeletal pattern associated with MLIs in an orthodontic population.

The objectives of this study were to investigate the prevalence of MLI agenesis in an orthodontic population, and to determine the prevalence of associated dental anomalies and skeletal malocclusions, to compare our results with published studies, and to report the treatment received by the subjects.

## Material and Methods

The clinical records (case histories, panoramic and full mouth periapical radiographs and study models) of orthodontic patients referred to the Department of Orthodontics, Ataturk University, between January 2004 and January 2009, were used to determine agenesis of MLI. If an accurate diagnosis of the agenesis could not be made from these records, the subject was excluded from the study. All subjects in this study were Caucasian and had no developmental anomalies such as cleft lip or palate, Down�s syndrome or ectodermal dysplasia. A total of 3872 subjects, between 12 and 25 years of age, were included in the study, and 27 subjects with developmental anomalies were excluded.

A tooth was diagnosed as agenesis if there was no evidence of crown calcification on the panoramic or full mouth periapical radiographs, and there was no evidence that it had been extracted. The case histories and study models were used to exclude subjects who had teeth extracted and to ensure an accurate diagnosis of MLI agenesis. The following data were recorded for each subject with agenesis of MLI: age; gender; unilateral or bilateral absence; anteroposterior skeletal relationship of the maxilla and mandible; presence of other dental anomalies such as a supernumerary tooth, dilacerated tooth, transmigrated tooth, lateral incisor-canine transposition, impacted and ectopically erupted maxillary canines; and the agenesis of other teeth, excluding the third molars. When one lateral incisor was absent the crown of the contralateral incisor was also analysed for reduced or peg-shaped form, using the criteria described by Albashaireh and Khader (17). Amount of the crowding prior to the orthodontic treatment and the mesio-distal diameters of the MLI for the presence of microdontia were measured using digital calipers (Mitutoyo, Tokyo, Japan).

The lateral cephalometric film of each subject with MLI agenesis was traced and the anteroposterior skeletal relationship of the maxilla and mandible classified as Skeletal Class I, II or III, depending on the size of the ANB angle. Angles between 0 and 4 degrees were Class I, > 4 degrees Class II and < 0 degrees Class III.

The data were analysed with chi-squared tests and the prevalence of MLI in this sample was compared with published data (13,18-24). The Statistical Package for Social Sciences (SPSS 12.0) was used and the significance level was p <0.05.

To determine errors in the methods, 10 per cent of the subjects with or without agenesis of MLI were selected randomly and evaluated by another researcher four weeks after the initial survey. The agreement between both investigators was 100 per cent. To determine the errors associated with digitizing and measurement of the ANB angle, 15 lateral cephalometric films of subjects with tooth agenesis were randomly selected and remeasured by the same author four weeks after the first set of measurements. The coefficients of reliability of the measurements were above 0.93 (25).

## Results

Of the 3872 subjects (2079 females, 1793 males) examined, 94 (61 females, 33 males) were found to have MLI agenesis. Thus, the prevalence of MLI agenesis in our sample was 2.4 per cent, and 2.9 per cent of the females and 1.8 per cent of the males were affected. The diffe-rence between the genders was statistically significant (x2 = 4.79; p < 0.05). Bilateral agenesis of MLI occurred in 52 subjects (55.3 per cent) and unilateral agenesis in 42 patients (44.7 per cent). Of those presenting with unilateral agenesis of the MLI, 30 (71.4 per cent) were on the right side and 12 (28.6 per cent) on the left side. No gender difference was observed in the side-to-side distribution of MLI agenesis (p > 0.05).

The characteristic features of the dental anomalies associated with MLI agenesis are given in  Table 1. These anomalies were: impaction, transmigration and ectopic eruption of maxillary canines, MLI-canine transposition, dilacerations, supernumerary tooth, and other missing teeth, excluding the third molars. Sixty-two of the subjects with the agenesis of MLI had some form of dental anomaly (66.0 per cent), while the remainder (34.0 per cent) had no dental anomalies. The most commonly observed dental anomalies associated with the agenesis of MLI were ectopic eruption of maxillary canines and reduced or peg-shaped MLIs with the frequencies of 21.3 per cent and 20.2 per cent, respectively. Of the 42 patients with unilateral MLI agenesis, the crowns of the contra-lateral incisor were modified or peg-shaped in 19 patients (45.2 per cent). When we excluded the 19 subjects with reduced crown or peg-shaped lateral incisors, 81.4 per cent (N = 35) of the remainder (N = 43) had a dental anomaly on the side with the MLI agenesis. In addition to these data, 85.5 per cent (N = 53) of the subjects with MLI agenesis had unilateral dental anomalies.

**Table 1:**
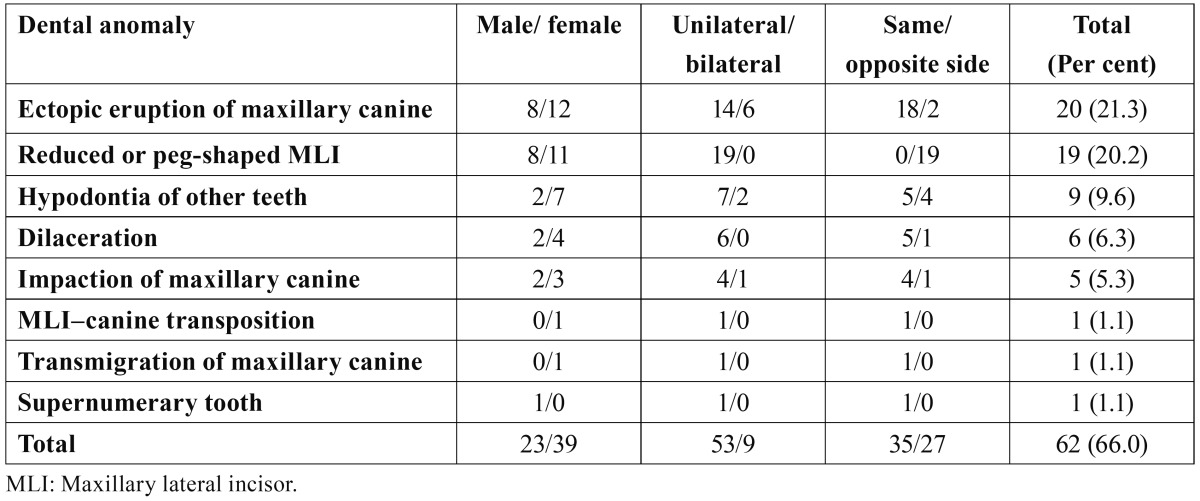
Dental anomalies found in subjects with maxillary lateral incisor agenesis.

The distributions of the skeletal classes in our sample and a reference study are given in  Table 2. Subjects with MLI agenesis had a significantly higher prevalence of skeletal Class III malocclusion than the reference study (p < 0.001). The frequencies of subjects with MLI agenesis in Class I, Class II and Class III malocclusions were, respectively 55.3 per cent, 16.0 per cent and 28.7 per cent.

**Table 2:**
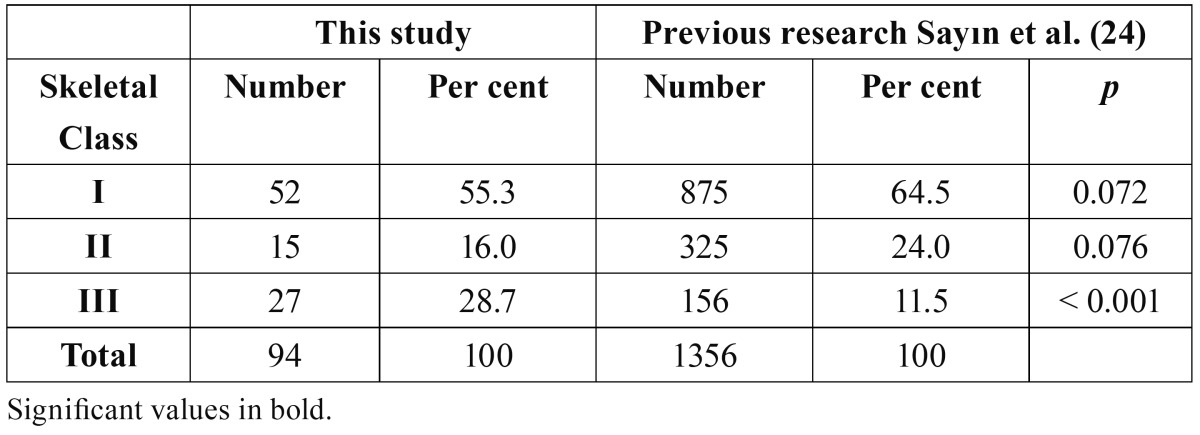
Prevalence of skeletal classes in subjects with maxillary lateral incisor agenesis.

The prevalence rates of dental anomalies associated with MLI agenesis in the present study are compared with several reference studies ( Table 3). The prevalence of ectopic eruption (p < 0.001), transposition (p < 0.05), and transmigration of the maxillary canines (p < 0.001) and reduced or peg-shaped MLIs (p < 0.001) were significantly greater in our sample as compared with published data of general populations. There were no statistically significant differences in the prevalence of supernumerary tooth (p = 0.903), dilacerated teeth (p = 0.336), agenesis of the other teeth (p = 0.058) and maxillary canine impactions (p = 0.541) in our sample as compared with the reference studies.

**Table 3:**
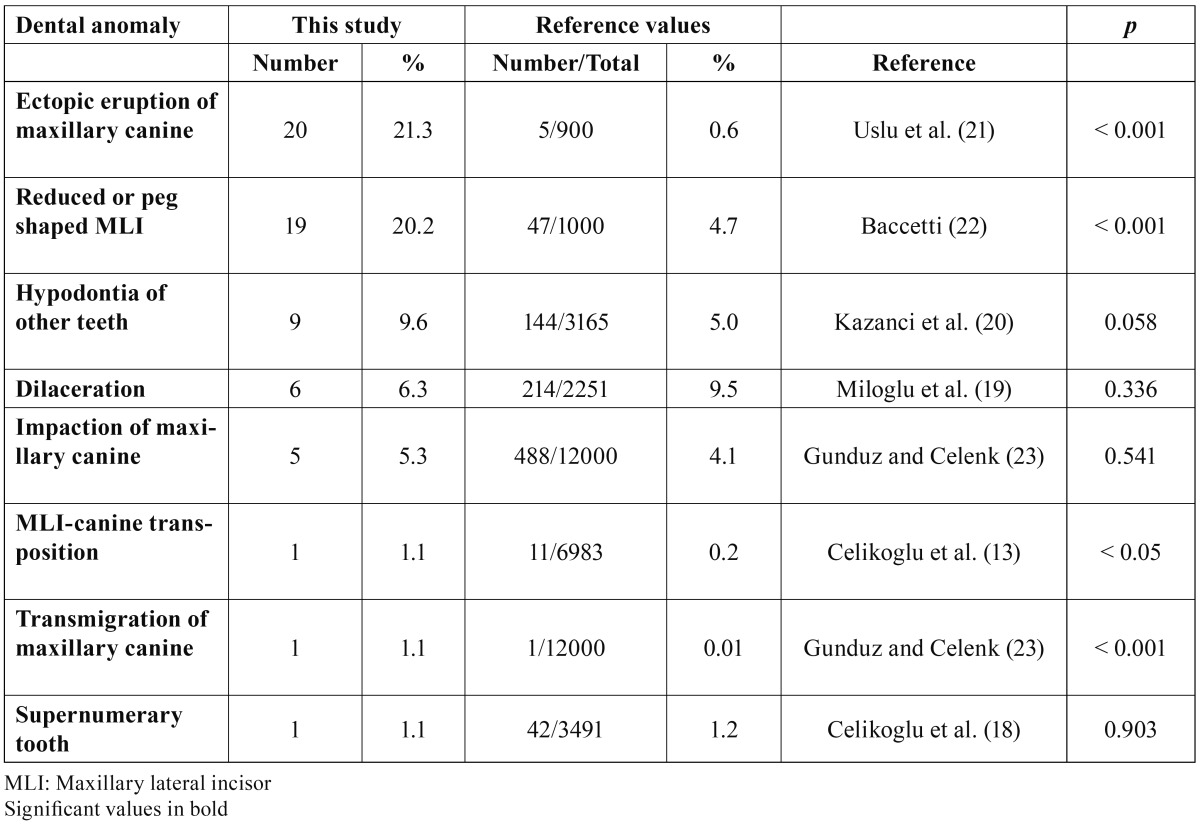
Comparisons of the prevalence rates of dental anomalies found in subjects with maxillary lateral incisor agenesis and previous research.

The MLI space was closed with fixed appliances in 78.7 per cent of the subjects and maintained/opened for a prosthetic restoration in the remainder. As a rule, the lateral incisor space was closed in the subjects with crowded arches and maintained or opened in the subjects with no crowding or spaced arches.

## Discussion

We investigated the prevalence of MLI agenesis in an orthodontic population and found that approximately 2 per cent of our subjects had agenesis of one or both maxillary incisors. Our finding falls in the lower end of the range (between 1 and 11 per cent) reported by others (4,5,9,15,26,27). It is impossible to determine the contributions made by ethnic factors and/or other factors because of the lack of information in the records we used and the factors influencing referral and eventual treatment of the subjects (all of our subjects were diagnosed with a malocclusion, sought and accepted orthodontic treatment) (10).

Unlike many other studies, we found a significantly higher prevalence of MLI agenesis in females (1,3-7,10,26). Our findings that both MLIs were just as likely to be missing as one incisor, and when one lateral incisor was missing it was likely to be on the right side, agree with previous researches (3,5,9,28). However, we urge caution when interpreting these results because of the methodological shortcomings in retrospective studies of orthodontic populations.

Previous studies (10-14,29) have shown that tooth agenesis may be related to other dental anomalies such as microdontia or peg-shaped incisors, taurodontism, transposition, supernumerary tooth, ectopic eruption, retained primary tooth, and ectopic eruption. However, agenesis of MLI and associated dental anomalies were limited in the literature. Most of the papers (5,9,17,28) published about MLI agenesis investigated reduced crown size or peg shaped form of the contralateral MLI among the subjects with unilateral absence of this tooth. Pinho et al. (5) investigated other associated developmentally absent teeth and supernumerary tooth. Although no supernumerary tooth was found, they found that 12.8 per cent of the subjects with MLI agenesis had absence of other teeth and most frequently observed missing teeth were maxillary and mandibular premolars. The prevalence of the subjects with agenesis of other teeth (9.6 per cent), in this study, was very close to the data reported by Pinho et al. (5) and the missing teeth were maxillary and mandibular premolars (63.6 per cent) and mandibular central incisors (36.4 per cent). As shown in table 1, agenesis was detected more commonly unilateral, in females, and in the same side with the MLI agenesis. The maxillary second premolar followed by the mandibular central incisors was the most frequently absent teeth. Celikoglu et al. (13) reported MLI-canine transposition in the cases of MLI agenesis and Peck et al. (11) showed transposition in the mandible. In this study, one subject with MLI-canine transposition in the same side with MLI agenesis was observed. Additionally, we found 6 subjects with dilacerations, 5 with the impaction of maxillary canines, 1 with a supernumerary tooth, and 1 with a transmigrated maxillary canine. Supernumerary tooth was an extra premolar in the same side with the MLI agenesis. In addition, transmigration and transposition of the maxillary canine were also in the same side with the MLI agenesis.

The most commonly observed dental anomaly associated with the agenesis of MLI was found to be ectopic eruption of maxillary canines with a prevalence of 21.3 per cent. Ectopic eruption occurred in the same side with MLI agenesis in 90.0 per cent (n=18) of the subjects and unilateral in 70.0 per cent (n=14) of the patients. In two cases, the ectopic eruptions were observed at the side of reduced or peg shaped MLI. In agreement with our data, Garib et al. (29) showed that most of the ectopic eruption of the maxillary canines was unilateral and at the same side with the MLI agenesis. On the other hand, Becker et al. (30) found that ectopic eruption of the maxillary canines occurred more frequently on the side of the reduced or peg shaped MLIs than on the side of the MLI agenesis. Hence, we can say that agenesis and microdontia of MLIs are the major etiologic factors of maxillary canine ectopic eruption.

Of the 42 patients who had unilateral absence, 19 (45.2 per cent) were found to have a microdont or peg shaped lateral incisor on the other side. Albashaireh et al. (17) demonstrated that there was a 50.0 per cent microdont or peg- shaped MLIs on the other side in the individuals with unilateral MLI agenesis. On the other hand, Stamatiou et al. (9) reported that 33.0 per cent of the patients had modified tooth form or peg shaped lateral incisors.

When comparing the prevalence rates of associated dental anomalies between the subjects with MLI agenesis and reference values (13,18-24), we found significantly increased prevalence rates for ectopic eruption, transposition, and transmigration of the maxillary canines and reduced or peg- shaped MLIs in the study sample. There was only one study (29) comparing the results with reference values; however, transposition, transmigration, ectopic eruption of maxillary canine, dilacerations, and impaction of the maxillary canine were not assessed in that study. In agreement with our findings, they found similar results for peg- shaped lateral and supernumerary teeth. The authors showed increased prevalence rates for agenesis of other teeth, however, we could not find statistically significant difference in the prevalence of tooth agenesis excluding third molars.

Although some reports (4,31) showed that the skeletal morphology of patients with tooth agenesis includes a tendency towards skeletal Class III pattern, the skeletal characteristics of tooth agenesis showed that patients with skeletal Class II were significantly less affected by tooth agenesis compared with the control group (1). Those studies investigated the anterior-posterior skeletal relationship of the maxilla and mandible in patient with agenesis of all permanent teeth. There was no study comparing the skeletal classes between the patients with MLIs and general population. Hence, this study seems to be the first. Patients with missing MLIs showed a significantly higher prevalence of skeletal Class III malocclusion (P < 0.001) when compared with the control group of general population (24).

In 78.7 per cent of the patients with the agenesis of MLIs, the space was orthodontically closed, while in the remaining 21.3 per cent the space was orthodontically maintained for prosthetic replacements and implant placement. The lateral incisor space was closed in the patients with crowded arches, while space was maintained in the patients with uncrowded arches. Since crowding was present in the study group and implant treatment is deferred until the jaws have stopped growing to avoid the complications caused by implants (32), the space was orthodontically closed in most of the patients. Robertsson et al. (32) investigated the aesthetics according to the opinions of the patients, occlusal function, and periodontal health in subjects with one or two MLI agenesis who had received either orthodontic space opening or closure followed by a modern prosthetic replacement for the MLI agenesis. The authors indicated that orthodontic space closure produced treatment results that appear to be reasonably stable, and better accepted by the patients than prosthetic replacements.

Orthodontic patients do not necessarily reflect the number of individuals in the population with tooth agenesis, this will be dependent on the availability of orthodontic treatment and its uptake in this particular population. However, retrospective studies rely on good record keeping and orthodontic patients often have more complete records (10). Thus, some reports (1,4,6,7,10,15,20,26) have shown the prevalence of tooth agenesis in orthodontic patients.
